# Body posture aftereffects—does viewing slouched bodies change people’s perception of normal posture?

**DOI:** 10.1098/rsos.241677

**Published:** 2025-03-26

**Authors:** Eva Tzschaschel, Ian D. Stephen, Kevin Brooks

**Affiliations:** ^1^ School of Psychological Sciences, Macquarie University, Sydney, New South Wales, Australia; ^2^ Lifespan Health and Wellbeing Research Centre, Macquarie University, Sydney, Australia; ^3^ Bournemouth University, Poole, UK

**Keywords:** adaptation, aftereffects, body image, body posture

## Abstract

People lead increasingly sedentary lifestyles and spend extended periods sitting in slouched and head-forward positions, which can lead to health issues. People are so accustomed to seeing slouched posture that they may perceive it as normal and fail to notice their own slouched posture. We aim to investigate this possibility using the visual adaptation paradigm, which has provided insights into the perception of body size and shape in the context of exposure to thin bodies in the media. The experiment was conducted in three phases. First, participants established the posture they perceived as normal by manipulating body stimuli shown in profile view. In the second phase, the adaptation phase, participants viewed bodies with extremely upright or slouched postures before establishing their perceived normal posture again in the third phase. Perceived normal posture differed significantly before versus after adaptation, demonstrating a visual aftereffect. However, this only applied if test and adaptation bodies were presented in the same orientation, suggesting that our representation of posture is retina-centred rather than object-centred. This result reduces the likelihood that visual adaptation influences the increase in slouched posture in the population. These results contribute to understanding visual influences on people’s perception of body posture.

## 1. Introduction

Maintaining a sedentary lifestyle can lead to poor posture habits, such as slouching, which can increase the likelihood of developing chronic health issues [1]. Sitting (or standing) for prolonged periods can cause low back pain, discomfort in the shoulders and neck, and overall musculoskeletal pain [2–4]. Research has shown that slouched posture is associated with increased symptoms of depression and anxiety, including lower self-esteem, negative mood and stress responses [5–7]. A typical body posture, hereafter referred to as a ‘normal posture’, is the natural alignment of the body so that the body can be held upright with minimal effort [8]. This stance features a mid-range pelvic position, slight lumbar lordosis (inward curve towards the centre of the body), slight thoracic kyphosis (outward curve away from the centre of the body) and a slight cervical lordosis of the spine [8–10]. Deviations from the normal posture can lead to musculoskeletal pain, which is a global problem affecting over 600 million people worldwide, costing the Australian health system $4.8 billion annually [11,12]. Jung *et al*. [13] have shown that increased smartphone use can have negative effects on posture, promoting further prolonged periods of slouched posture. People may be so used to seeing slouched posture that they perceive it as normal, failing to notice if their own posture is slouched. Increasing awareness of slouched posture and improving our understanding of how posture is perceived and processed in the brain is vital to preventing or correcting slouched posture.

To comprehend how we are affected by viewing the posture of others, we must first examine how our visual system perceives our environment. This system does not simply give us a veridical representation of the real world. Instead, context and prior exposure can affect the usual neural processes of vision, leading to perceptual bias. For example, visual adaptation refers to a change in neural activity experienced due to prolonged exposure to a particular stimulus feature. The consequences of this exposure and adaptation include a perceptual bias called an aftereffect [14]. There are many examples, such as the motion aftereffect described by Robert Addams in 1834. After observing a waterfall for some time, he looked at the neighbouring rocks and reported that they appeared to move upward [15].

Perception research has identified many other forms of visual adaptation. For example, there are aftereffects of colour [16], line or edge orientation [17] and curvature [18], among many others. In the context of this study, the curvature aftereffect may be particularly relevant. When participants are exposed to lines that are curved slightly (the ‘adaptation’ stimulus, or ‘adaptor’), a subsequently shown curved line (the ‘test’ stimulus) will look straighter than it is, with objectively straight lines appearing curved in the opposite direction [18]. Adaptation to a slouched body posture could produce similar results, such that viewing the slouched postures of other individuals could result in them being perceived as normal. Thus, this could encourage these observers to adopt a more slouched posture.

It is important to note that adaptation has been demonstrated for stimuli such as faces and bodies. For instance, exposure to distorted faces can cause normal faces to appear distorted in the opposite direction [19,20]. In body adaptation research, exposure to body stimuli can influence people’s perceptions of their bodies and those of others. For example, after participants viewed large bodies in the adaptation phase, images of normal-sized bodies appeared thinner than they were. Conversely, bodies appeared larger after participants had adapted to thin bodies [21].

In categorizing visual adaptation effects, a distinction between low and high-level adaptation is often made [22]. Low-level adaptation affects the response properties of neurons activated early in the visual processing hierarchy, responsible for processing relatively simple stimulus attributes, such as orientation. Given that these neurons respond to stimuli located within a very small area of the retina, the adaptation and test stimuli must be presented at exactly the same retinal location for an effect to be demonstrated. As such, these effects have a clear retinal frame of reference [23]. The aftereffects mentioned earlier of orientation, motion and colour all have these low-level properties. In contrast, high-level adaptation is thought to occur in neural regions that are activated later and encode more complex stimulus properties, such as the apparent gender of a face or the shape of a body. Consistent with this proposal, previous research has demonstrated that adaptation of facial configuration and body size is high-level [24–26]. In particular, Brooks *et al*. [27], inspired by a previous face adaptation study [26], showed that body size adaptation operates in an object-centred rather than a retinal frame of reference. When the adaptation and the test stimuli differed in orientation (i.e. were tilted at +45 and −45°), the aftereffect was not fixed on the retina but rotated with the body stimulus. Given that other body aftereffects show this property, it seems plausible that posture adaptation, if demonstrated, may also operate in an object-centred frame of reference.

This study examines whether visual exposure to extreme postures can produce a visual aftereffect and whether this aftereffect operates in a retinal frame of reference (as for low-level effects) or an object-centred frame of reference (as for high-level effects). Using stimuli depicting bodies viewed in profile, we present adapting and test stimuli facing the same direction (congruent) or in opposite directions (incongruent). We hypothesize that aftereffects will be shown in both the congruent and incongruent conditions. Demonstrating an aftereffect in the congruent case would constitute evidence of adaptation for the perception of posture. An aftereffect in the incongruent condition would suggest these processes operate in an object-centred frame of reference. To the best of our knowledge, this study is the first to attempt to establish an aftereffect of posture, setting a starting point for research in this area.

## 2. Method

### 2.1. Participants

An *a priori* power analysis using G*Power 3.1.9.2 [28] indicated a minimum sample size of 84 to give 95% power to detect a small-medium effect for the interaction term, which is the key test of our hypothesis. Participants were recruited from two sources. The first group was students (*n* = 102) from a first-year undergraduate research participation pool at Macquarie University who participated for course credit. The remaining participants (*n* = 16) were members of the public recruited via social media and word of mouth who were not compensated for their participation. We excluded seven participants who failed the attention check during the adaptation phase twice (of six attention checks). Of the remaining 111 participants, 76 (68.5%) identified as female, 31 (27.9%) as male and 4 (3.6%) as non-binary. The mean age was 23.1 years (s.d. = 10.3), and over half of the participants reported their ethnicity as European/White (53%). The remaining ethnicities were East Asian (15.3%), South Asian (13.5%), Middle Eastern (9%) and other (7.2%), while two participants declined to answer (1.8%). Participants completed the study online via the Gorilla Experiment Builder software (https://www.gorilla.sc/) between June and September 2023. Completing the study took approximately 30 min.

### 2.2. Design

The study used a 2 × 2 mixed ANOVA. The between-subjects variable was Adaptation Posture, with two levels: slouched and upright. The within-subjects variable was congruency between the test and adaptor view, with two levels: congruent (i.e. facing the same way) and incongruent (i.e. facing the opposite direction).

The dependent variable was a change in the point of subjective normality (ΔPSN) for posture. The point of subjective normality (PSN) indicates the posture that a participant perceives as normal, averaged across all test stimuli both before (baseline test) and after (adaptation test) exposure to the adaptation stimuli. High PSNs indicate that upright postures appear normal, while lower PSNs mean more slouched postures are considered normal. Changes in PSN from baseline to adaptation are calculated by subtracting the baseline test score from the adaptation test score. Negative scores indicate that adaptation has caused an aftereffect such that stimuli appear more upright than they really are, and hence, more slouched bodies are perceived as normal. Conversely, positive scores indicate an aftereffect wherein stimuli appear more slouched after adaptation, and hence, more upright bodies are perceived as normal.

### 2.3. Stimuli

#### 2.3.1. Stimulus creation

Stimuli were created from photographs of 58 male volunteers from a previous study [29]. These photographs were taken using a Canon 50D digital camera with a 1/50 s exposure time, a lens aperture of F/5.6, a white balance set at 6500K and an ISO speed rating of 200. Subjects stood in a photo booth painted Munsell N5 neutral grey, lit by 15 high-accuracy d65 fluorescent tubes in high-frequency fixtures to reduce flicker effects. Perspex diffusers were used to ensure even light distribution. Each identity was photographed wearing grey shorts and a grey singlet, in left profile, once with a slouched and once with an upright standing posture. Faces and heads were pixelated in Photoshop (version CC 2018; Mosaic, size 32) to avoid face adaptation.

**Figure 1.**
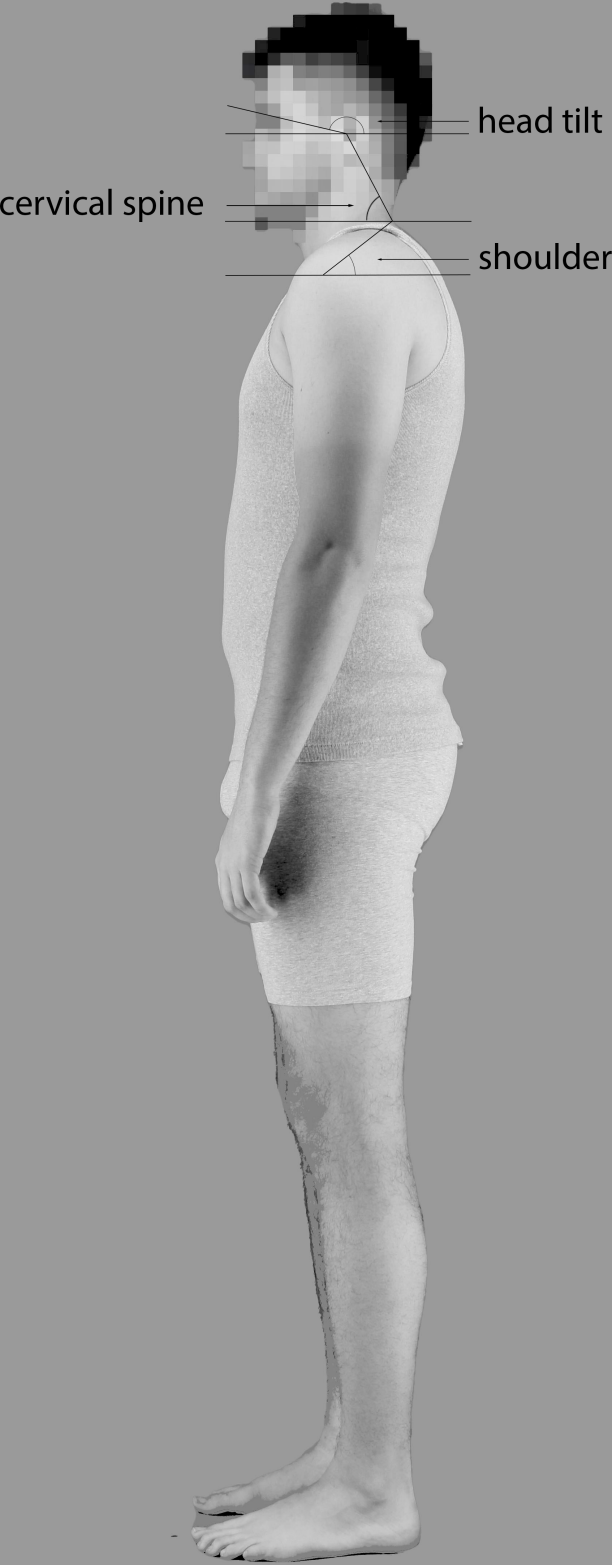
Illustration of the three angles used for determining upright and slouched posture (cervical spine, head tilt and shoulder; adapted from Raine & Twomey, [30]).

We measured the postures of the 58 identities using head tilt, cervical spine and shoulder angles [30] (see figure 1). From these identities, we chose 10 with the most upright and 10 with the most slouched posture.

**Figure 2.**
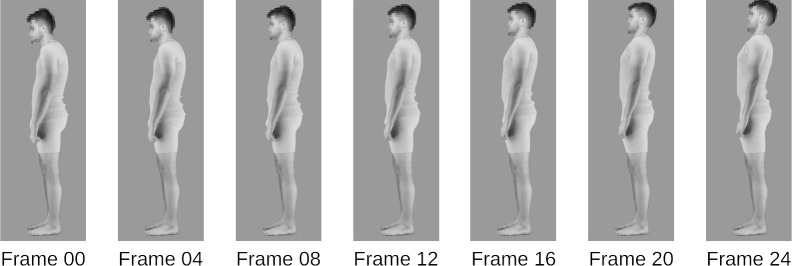
Examples of every fourth frame are from the extreme slouched position (frame 00) to the extreme upright position (frame 24).

**Figure 3.**
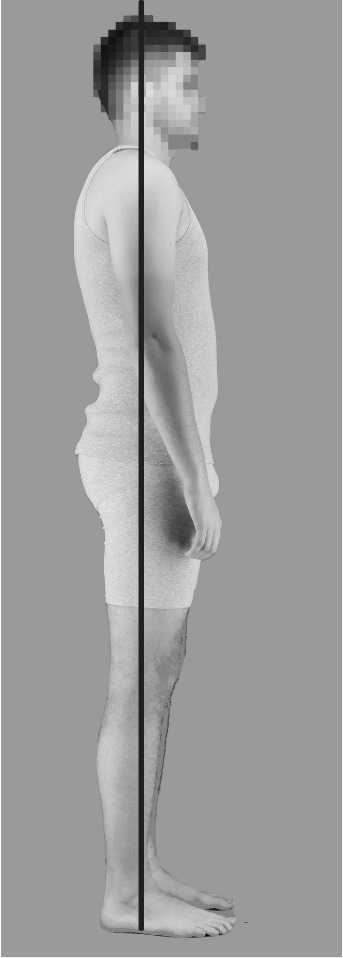
Gravity line measure for ideal posture (Image adapted from Munivrana *et al*. [32]).

We averaged these two groups of 10 bodies to create a slouched and upright body prototype using the image-morphing software Webmorph [31]. These allowed us to isolate the differences between more upright and slouched postures, independent of other image aspects. Using these prototypes, we created a series of 25 interpolated postures for each identity (figure 2), from extremely slouched (frame 00) to extremely upright (frame 24). Frame 12 of each identity was the closest to the gravity line measurement of ideal posture, where landmarks such as the ear, shoulders, hips, knees and ankles are aligned in a single, vertical line in the sagittal plane (figure 3).

After morphing, we excluded four identities due to image distortions, leaving 16 identities for the experiment. Two identities were selected for the practice task for all participants, whereas the remaining 14 identities were used for both test and adaptor stimuli. To avoid potential confounding effects of identity, four versions of the study were created, each of which had six identities randomly allocated to be adaptation stimuli and the remaining eight as test stimuli. Participants were then randomly allocated to one of these four versions.

#### 2.3.2. Test stimuli

The 25 frames described above were presented, one at a time, on the computer screen, and participants were asked to use the p and q keys to make the body ‘look as normal as possible’. Pressing the q key caused the presented frame to be replaced by the next most slouched in the sequence, while pressing the p key caused the presented frame to be replaced by the next most upright in the sequence. The small increments separating the 25 postures ensured a perceptually smooth transition. The sequence was looped to avoid participants being able to identify the endpoints of the sequence. Participants could cycle through the frames as much as they wished before clicking a button on the bottom of the screen labelled ‘Select’ to save their choice of most normal posture.

Depending on the condition, test stimuli could be displayed in either left- or right-profile views. The right profiles involved a mirror-image version of each body created in Photoshop. The test stimuli were presented at the centre of the screen, with the size determined by the Gorilla Experiment Builder software (www.gorilla.sc), which guarantees that objects are displayed with the correct aspect ratio regardless of the size and aspect ratio of the participant’s screen. To ensure this technology functioned effectively for all participants, we did not allow mobile phones or tablets and ensured that participants viewed stimuli in full-screen mode.

#### 2.3.3. Adaptation stimuli

For the adaptation stimuli, we exclusively used the extreme posture images, i.e. frames 0 (extremely slouched) and 24 (extremely upright) described above (figure 2). Depending on the condition, the adaptation stimuli were shown in either a left or right profile view and randomly presented in nine positions arranged in a square centred in the middle of the screen. Adaptation images were 71.2% of the size of the test stimuli to minimize retinotopic low-level adaptation effects.

### 2.4. Procedure

#### 2.4.1. Consent and demographics

Participants started the study by giving informed consent and answering demographic questions regarding their age, gender and ethnicity.

#### 2.4.2. Practice phase

During the practice phase, which was identical to the test phase, participants familiarized themselves with the process of manipulating the posture of the two practice identities.

#### 2.4.3. Baseline test phase

During the baseline test phase, participants were shown stimuli in both the left and right profile views in separate trials. In each trial, participants pressed nominated keys (p and q) on their computer keyboards to manipulate the frames to their desired final position. They clicked the ‘select’ button to establish their PSN. This process was repeated for eight test identities randomly selected from the pool of 14 identities, four facing left and four facing right, each shown twice, for a total of 16 trials.

#### 2.4.4. Adaptation test phase

During this phase, depending on the condition the participant had been allocated to, we displayed adaptation bodies with either a slouched or an upright posture and in either right-facing or left-facing profile views. However, as in the baseline test phase, two views of test stimuli were displayed to all participants—in both the left and right profiles. Hence, regardless of whether a participant saw adaptors facing to the right or the left, some test stimuli would be seen in a view congruent with the adaptors, while others would have been incongruent.

In the initial adaptation sequence, participants viewed six adaptors 10 times for 2 s each, for a total initial adaptation duration of 2 min. We included attention checks to ensure participants viewed the screen continuously during the adaptation phase as instructed. The words ‘Press the space bar’ appeared below the image for six randomly selected adaptation stimulus presentations for 2 s. Participants who failed to comply twice or more were excluded from the study.

#### 2.4.5. Post-adaptation phase

Immediately following initial adaptation, participants established their post-adaptation PSN using the same task as in the baseline test. We used top-up adaptation between each trial, presenting each of the six adaptation identities for 2 s each, making each top-up sequence 12 s long.

**Figure 4.**
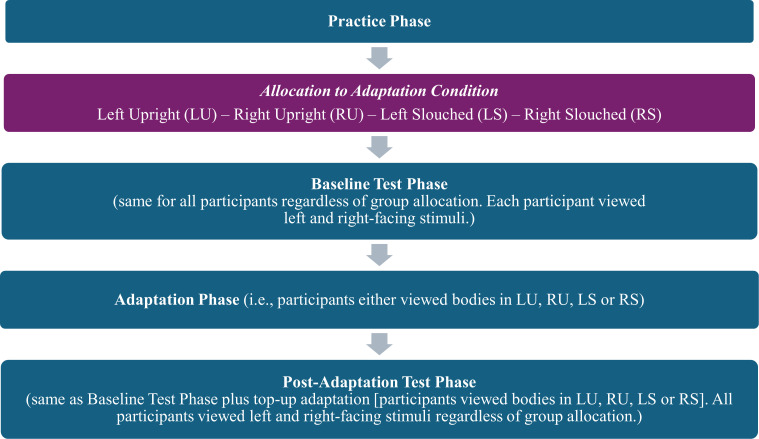
Study flow diagram.

A schematic sequence of the procedure, including the randomizations we used to avoid systematic effects from both stimuli and participant characteristics, can be viewed in figure 4.

## 3. Results

We first analyzed participants’ perceptions of normal posture at baseline. Participants chose a posture that was slightly (*M* = 14.43, s.d. = 3.07) more upright than the natural (unmanipulated) posture of the subjects depicted in the stimulus photographs (one-sample *t*‐test, *μ* = 12, *t* = 15.84, *p* < 0.001). An independent samples *t*‐test showed no significant difference in baseline PSN between those later adapted to upright (*M* = 14.14, s.d. = 1.91) versus slouched (*M* = 14.55, s.d. = 2.13) posture (*t*(108) = 1.06, *p* = 0.291, *d* = 0.20).

**Figure 5.**
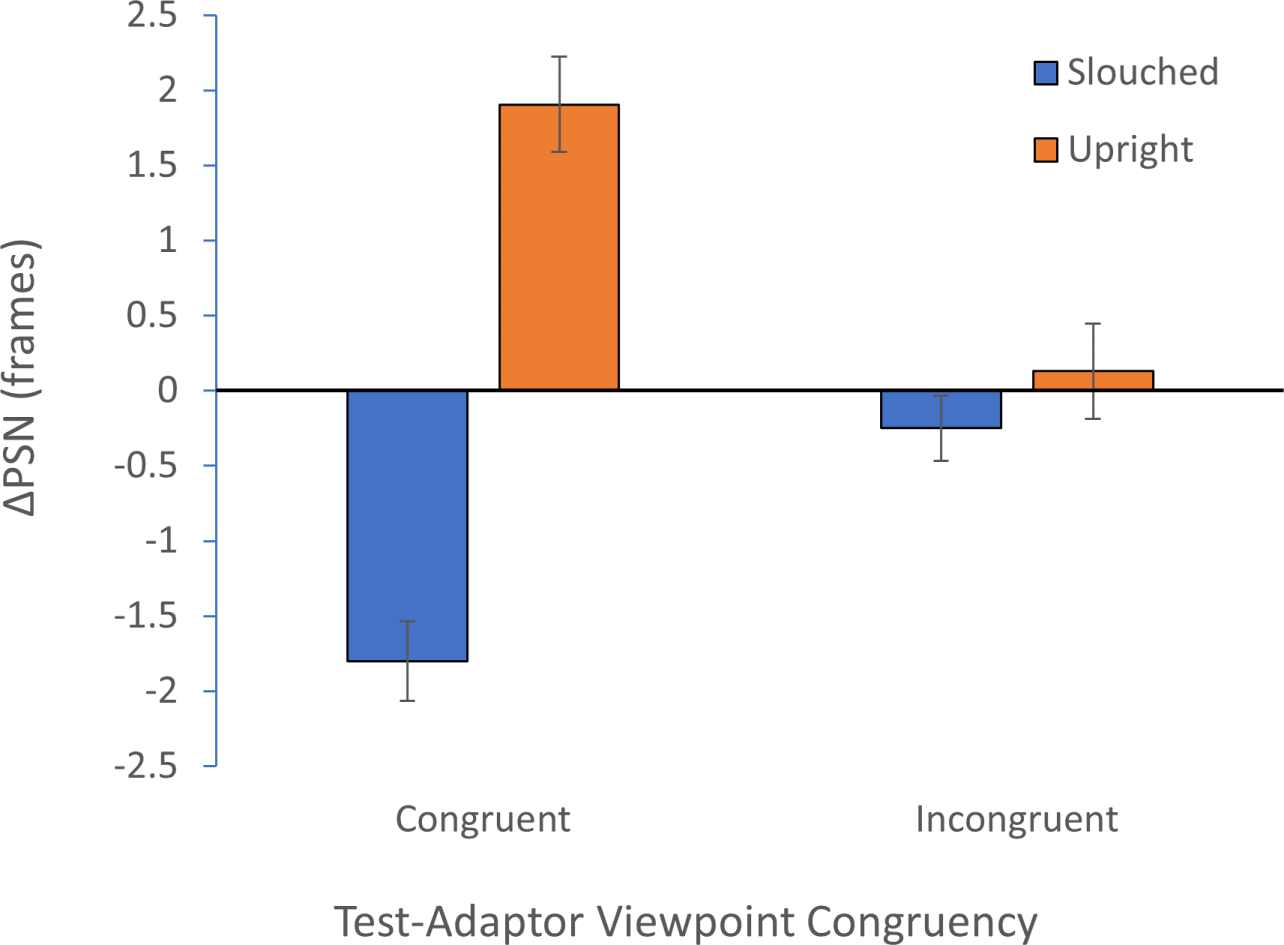
Graph showing the ΔPSN (mean, standard error of mean [SEM]) in the four conditions: blue bars show slouched conditions, and orange bars show upright conditions.

We further conducted analyses pertinent to our main hypotheses. The effects of posture adaptation on perceived body posture were measured by the ΔPSN in congruent and incongruent conditions, as shown in figure 5. From informal inspection, in the congruent conditions, we can observe a negative ΔPSN after adaptation to slouched bodies and a positive ΔPSN after adaptation to upright bodies. However, for the incongruent conditions, ΔPSN values were similar and near zero for both slouched and upright adaptation.

The statistical significance of these general observations was formally assessed using a 2 × 2 mixed ANOVA. The main effect of adaptation posture was significant, *F*_1,108_ = 32.90, *p* < 0.001, *ƞ_p_^2^*p20.23. There was also a significant interaction between congruency and posture, *F*_1,108_ = 79.33, *p* < 0.001, *ƞ_p_*^2^p20.42. The main effect of congruency was not significant, *F*_1,108_ = 0.36, *p* = 0.548, *ƞ_p_*^2^*p*2.00.

These effects were further examined using four planned one-sample *t*-tests, one for each condition. We used the Holm–Bonferroni method, a stepwise procedure for controlling the family-wise error rate. The ΔPSN means for the congruent slouched (*M* = −1.8, s.d. = 3.5, *t*_395_ = −10.19, *p* < 0.001) and Congruent Upright conditions (*M* = 1.9, s.d. = 3.8, *t*_425_ = 10.43, *p* < 0.001) were significantly different from zero after applying the Holm–Bonferroni correction (alpha values of 0.0125 and 0.0167, respectively). Although both data sets for the incongruent conditions showed differences from zero in the predicted directions, neither was significant (incongruent slouched: *M* = −0.2, s.d. = 3.2, *t*_(398)_ = −1.44, *p* = 0.150; incongruent upright: *M* = 0.2, s.d. = 3.8 *t*_(428)_ = 0.81, *p* = 0.418).

## 4. Discussion

The results confirm our hypothesis that participants would visually adapt to body posture. To the best of our knowledge, this is the first report of a visual aftereffect of body posture. Specifically, after visual exposure to slouched bodies, participants perceived test stimuli as more upright. Consequently, when asked to set posture to appear normal, the aftereffect led them to adjust their bodies to be objectively more slouched. Conversely, after adapting to upright bodies, participants perceived test stimuli as more slouched, leading them to select more upright bodies as appearing normal. Adaptation to extreme body postures produces an aftereffect on perceived posture. However, contrary to our second hypothesis, this aftereffect was not significant when adaptors and test stimuli were incongruent in terms of their viewpoint angle. We would expect aftereffects operating within an object-centred frame of reference to be determined by the body posture (regardless of the viewpoint from which the body is seen) and not the specific direction of the body’s curve. Hence, such effects should transfer across viewpoints. Conversely, low-level effects would be determined by the curvature in a specific direction and should not transfer across viewpoints. Our results showed adaptation in the congruent but not incongruent condition, which aligns with our predictions for the retina-centred nature of representation but not with prediction for an object-centred frame of reference.

Brooks *et al*.’s [27] findings on body size adaptation led us to hypothesize that exposure to extreme body postures would result in an object-centred aftereffect. Rotating test stimuli versus adaptors within the coronal plane by 90° allowed Brooks *et al*. [27] to observe the degree of aftereffect transfer between congruent and incongruent test–adaptor orientations. As our experiment used stimuli viewed in profile rather than in frontal view, we adapted their method by rotating our test stimuli versus adaptors within the transverse plane, resulting in the test stimuli and adaptors facing either the right or the left. Although our study aimed to minimize retina-based frame of reference adaptation effects by using different adaptation stimulus sizes compared to the test stimuli, our results in the incongruent condition indicate that the aftereffect on body posture works in a retina-based frame of reference.

Given the lack of evidence of an object-centred frame of reference, posture adaptation may reflect a more basic effect of orientation or curvature adaptation tied to retinal coordinates, i.e. a low-level effect [18]. Neurons that mediate the perception of orientation are located early in the visual cortex, in areas such as V1 and V2 [33]. Recalibration of these neurons’ response properties through adaptation is purported to produce low-level orientation aftereffects. Similarly, neurons responsible for the curvature perception are thought to be in area V4, a brain region higher in the visual system, which could also manifest low-level aftereffects [34].

However, we may need to consider that retina-centred and object-centred sensory structures could work together to interpret a stimulus [35]. Processing happens hierarchically in one direction from the retina to the higher visual brain regions, and feedback mechanisms could be employed to feed information back to lower regions. In the current study, in the congruent conditions, where test stimuli and adaptors face the same direction, any object-centred and retina-centred aftereffects could be considered additive to one another, working in the same direction to produce a more extreme ΔPSN. In contrast, in the incongruent conditions, object-centred and retina-centred adaptation would tend to cancel each other out. For example, consider a participant exposed to slouched adaptation stimuli facing to the right (curved to the right). This participant would show object-oriented adaptation such that more slouched bodies would appear more normal, regardless of the direction they are facing. They would also experience retina-oriented adaptation such that more slouched bodies would appear more normal if facing to the right (congruent condition). However, more upright bodies would appear more normal if facing to the left (incongruent condition). In the congruent condition, these two aftereffects would sum up, resulting in a large observed aftereffect. Conversely, in the incongruent condition, the two aftereffects would oppose each other, resulting in a smaller or possibly undetectable observed aftereffect. Therefore, our results could represent a combination of retina- and object-centred after-effects that operate in concert or conflict with one another. Future research should explore how the cortex encodes visual stimuli related to body posture and whether these processes occur in concert or conflict. A limitation of our study was that the body stimuli depicted male subjects and not female bodies. Stimuli were created from photographs of 58 male volunteers from a previous study [29]. Male stimuli were used due to concerns over non-specific responses to female profile stimuli. Future studies could examine whether these concerns are warranted.

## 5. Conclusion

Overall, our results suggest that adaptation to body posture is retina-oriented, making it less likely to serve as a mechanism for normalizing the misperception of body posture in the real world. However, it still appears that slouched posture is becoming more common and is being normalized in society [36,37], perhaps through mechanisms of social learning [38]. It is, therefore, essential to conduct ongoing and comprehensive research to understand the detrimental effects of poor posture due to prolonged periods of inactivity, starting with research into how our perception of those around us can determine how we prescribe normality to this important attribute. Further research into the broader implications of this issue and potential preventative measures is needed to effectively address the substantial economic and societal repercussions associated with the treatment of back pain, a leading cause of disability. This study contributes to this body of research by examining potential visual influences on people’s perception of body posture through adaptation. Although our conclusion that posture adaptation is primarily retina-based makes it less likely that visual adaptation underlies the increase in slouched posture in the population, these results offer important initial insights into how the brain perceives visual stimuli of body posture.

## Data Availability

Data available from the Dryad Digital Repository [39].
